# A Comparison of Piperacillin-Tazobactam With Conventional Antibiotics for Prophylaxis in Lower Extremity Type III Open Fractures

**DOI:** 10.7759/cureus.90890

**Published:** 2025-08-24

**Authors:** Anthony Paterno, Alysa Nash, Bradley Lauck, Anne Lachiewicz, Feng-Chang Lin, Robert Ostrum

**Affiliations:** 1 Orthopaedics, University of North Carolina at Chapel Hill, Chapel Hill, USA; 2 Internal Medicine - Infectious Diseases, University of North Carolina at Chapel Hill, Chapel Hill, USA; 3 Biostatistics, University of North Carolina at Chapel Hill, Chapel Hill, USA

**Keywords:** infection, open fracture, orthopaedics, prophylactic antibiotics, trauma

## Abstract

Background

Prophylactic antibiotics for Gustilo-Anderson type III open fractures are cefazolin plus gentamicin (CG) at many institutions. Adverse effects such as nephrotoxicity make gentamicin a less appealing drug, particularly in trauma patients. The purpose of our study was to compare piperacillin-tazobactam (PT) with conventional antibiotics in preventing surgical site infections (SSIs) in lower extremity type III open fractures.

Methods

This was a single-center, retrospective analysis of 164 patients with lower extremity type III open fractures at the University of North Carolina at Chapel Hill, a large academic level one trauma center in Chapel Hill, North Carolina. They were treated with three different antibiotic regimens that comprised the cohort. Patients were separated into three groups: those receiving PT, CG, or cefazolin plus gentamicin plus injected tobramycin (CGT), depending on current protocols or the surgeon's discretion. Primary outcomes are deep (requiring return to OR) and superficial (requiring antibiotics only) SSIs, as well as nonunions, during a postoperative period of 12 months.

Results

Our retrospective analysis from 2008 to 2019 revealed 164 type III open fractures. Seventy-two fractures were treated with PT, 44 with CG, and 48 with CGT. There was not a statistically significant difference in deep or superficial infections among the three groups (p = 0.41, p = 0.52, respectively). Nonunion rates were also not significantly different.

Conclusions

The results of this study demonstrate no significant difference in SSIs or nonunion rate between PT, CG, and CGT when used for infection prophylaxis in type III open fractures. We believe prophylaxis with PT may be equivalent to traditional regimens, potentially with fewer adverse effects, particularly in polytraumatized patients.

## Introduction

The Gustilo and Anderson classification is arguably the most widely accepted classification system for open fractures [1,2]. Type III fractures, as defined by Gustilo and Anderson, are those with >10 cm soft tissue disruption or high-energy injuries resulting in segmental long bone fractures or significant soft tissue degloving (IIIA), those requiring flap coverage (IIIB), or those with vascular injury requiring operative repair (IIIC) [1]. Surgical site infections (SSIs) are common complications in all open injuries, with type III fractures having the highest incidence of SSIs, reported between 10% and 50% in existing trauma literature [2-8]. This is attributed to the high-energy mechanisms that often produce these fractures and the subsequent extensive soft tissue and bony injuries.

Time to antibiotic administration following an open fracture is of the utmost importance, with an increased time associated with higher rates of infection [9-13]. Specific antibiotic protocols, however, are not as widely adopted. There is little consensus on the optimal antibiotic regimen for type III open fractures in particular. In addition, there is poor adherence to open fracture prophylactic antibiotic protocols that do exist [14]. Gustilo’s landmark study in 1976, which analyzed 352 open long bone fractures cultured at the time of presentation, recommended first-generation cephalosporins as the antibiotic of choice in open fractures [1]. This practice was widely accepted and supported by prospective trials and retrospective/systematic reviews in the literature [3,11,15]. A subsequent evaluation of all open fractures between 1976 and 1979 by Gustilo noted a shift in bacterial pathogen in infected open fractures to predominantly gram-negative organisms (77% from 24%) [2]. This prompted a recommendation of additional gram-negative coverage in the form of an aminoglycoside or third-generation cephalosporin in the instance of type III fractures [15]. Since this time, general practice has been to initiate a first-generation cephalosporin in type I and II fractures, with the addition of an aminoglycoside or third-generation cephalosporin in type III fractures.

The use of aminoglycoside antibiotics does not come without risk of complications. Nephrotoxicity is well described in the orthopaedic literature as a side effect of aminoglycosides [16-18]. This was further demonstrated by Bankhead-Kendall et al., who studied antibiotic prophylaxis in open fractures and saw a significant increase in the incidence of acute kidney injuries (AKIs) in patients receiving an aminoglycoside in addition to a cephalosporin [19]. Due to this side effect profile, some centers have looked to other regimens for prophylaxis of type III fractures. The purpose of this study was to assess the incidence of SSIs following the use of piperacillin-tazobactam (PT) monotherapy as antibiotic prophylaxis for type III open fractures. We hypothesized there would be no difference in SSI in the PT group compared to more traditional regimens of cefazolin and gentamicin (CG), as well as cefazolin, gentamicin, and local tobramycin (CGT).

## Materials and methods

After receiving institutional review board approval (approval no. 16-2689), we conducted a retrospective chart review of all patients with type III open fractures at the University of North Carolina at Chapel Hill, a large academic level one trauma center in Chapel Hill, North Carolina, from January 2008 to December 2019. We queried current procedural terminology (CPT) codes for lower extremity fractures, which included femoral shaft intramedullary nail (CPT 27506), distal femur open reduction internal fixation (ORIF) (CPT 27511, 27513), tibial plateau ORIF (CPT 27535, 27536), ORIF tibia shaft (CPT 27759), and ORIF of the ankle and pilon fractures (CPT 27792, 27814, 27822, 27827, 27828). Using operative and orthopaedic consult notes in the electronic medical record (EMR), a single investigator queried which of these were type III open fractures. Injury classification was ultimately confirmed by the documentation of the attending surgeon in the operative note of the initial debridement. Using the attending surgeon's operative reports keeps the definition of type III open fracture consistent, given the use of a single reviewer. No periprosthetic fractures were included in this study.

All patients included in this study received antibiotic prophylaxis according to the standard of care protocol in place at our institution at the time of presentation. For many years, the standard of care at our institution for antibiotic prophylaxis for type III open fractures was CG. Some surgeons at this time injected an aqueous solution of tobramycin into the wounds and added a vacuum dressing, using a cefazolin plus gentamicin plus injected tobramycin (80 mg tobramycin powder dissolved in 40 ml sterile saline) (CGT) treatment plan [20]. Cefazolin was dosed at 2 g every eight hours. Gentamicin was dosed based on trough data in conjunction with the pharmacy team. In June 2016, after approval by the Pharmacy and Therapeutics Committee, there was an institutional change to PT (piperacillin-tazobactam) monotherapy as the prophylactic antibiotic in type III fractures. PT was dosed at 3.375 g every six hours for only 24 hours after definitive fixation using an order set in our EMR. All other factors were kept consistent across the study groups. Therefore, we compared three prophylaxis antibiotic regimens: PT, CG, and CGT. Inclusion criteria were all skeletally mature patients with a type III lower extremity injury at our institution during our collection window. Patients who did not receive appropriate antibiotics for their injuries or who received a regimen that differed from our three study groups, as well as those with follow-up less than 12 months, were excluded. All patients received systemic antibiotics as soon as possible upon presentation, which were continued up to 24-72 hours following definitive fixation, the exact timing dictated by surgeon preference. 

The primary outcome was postoperative SSI. Using the fracture-related confirmatory and suggestive criteria established by Metsemakers et al. for fracture-related infection [21].We reviewed all patients' charts, looking for superficial and deep infection based on these parameters. Deep SSI is defined as those requiring a return to the operating room as well as treatment with antibiotics, while meeting the confirmatory criteria, and superficial SSI is defined as those treated with oral antibiotics alone and suggestive criteria [21]. Incidence of nonunion was also analyzed among the groups as a secondary outcome. Nonunion was defined by the treating surgeon per their clinic documentation. In all cases of nonunion in our series, a return to the operating room to address the nonunited bone was required. All patients were followed for at least 12 months postoperatively to ensure accurate nonunion diagnoses. 

Statistical analysis

When appropriate, descriptive statistics such as means, standard deviations, frequencies, and percentages were used to describe the patient’s demographic characteristics and clinical factors. The three antibiotic groups in this study were then analyzed for differences based on the analysis of variance (ANOVA) if the variable is continuous and normally distributed, and chi-square tests if the variable is categorical. An analysis of infection between patients with DM and ESRD was performed and compared to those patients without these comorbidities. Time to wound coverage and flap type were assessed as they related to deep infection. A nonparametric test, such as the Kruskal-Wallis test, was used if the variable is continuous but has a skewed distribution. The outcome variables, such as fracture pattern, Gustilo-Anderson classification, infection, and nonunion, were compared between antibiotic groups using chi-square tests of independence or Fisher’s exact tests if the chi-squared approximation may be incorrect. Statistical significance was set at p < 0.05. Prism statistical analysis software (Boston, MA) was used to perform the analysis. 

## Results

Using the EMR system, our CPT query yielded 5,587 total fractures. After manual screening, 239 of these were identified as type III open fractures. Seventy-five (31%) were excluded: 59 did not receive appropriate antibiotics for their injuries based on our institutional guidelines as described previously or did not fit into one of the three groups, nine had insufficient follow-up, and one had an amputation in the immediate postoperative period. This left 164 fractures included in the study, 48 (29%) in the CGT group, 44 (27%) in the CG group, and 72 (44%) in the PT group. The mean age among all groups was 42.1 ± 17.5, the mean BMI was 30.5 ± 9.1, and 70% of patients were male. Of all patients, 16% were known diabetics. None of these demographics reached statistical significance with respect to the different antibiotic groups compared. In addition, our cohort showed no difference in smoking history or the use of intramedullary nails or plates for operative fixation (Table 1). Forty-one percent of the injuries were tibial shaft fractures. Ankle/pilon fractures were the next most prevalent injury, representing 29% of the total fractures, while distal femur fractures represented 16% of the total (Table 2).

**Table 1:** Demographics by antibiotic group BMI: body mass index, ORIF: open reduction internal fixation, ESRD: end-stage renal disease

	Cefazolin plus gentamicin plus tobramycin (N = 48)	Cefazolin plus gentamicin (N = 44)	Piperacillin-tazobactam (N = 72)	Total (N=164)	P-value
Age	42.3 ± 17.5	41.2 ± 17.5	42.5 ± 17.7	42.1 ± 17.5	0.93
Gender					0.52
Male	34 (71%)	28 (64%)	53 (74%)	115 (70%)	
Female	14 (29%)	16 (36%)	19 (26%)	49 (30%)	
BMI	30.7 ± 11.1	31.0 ± 8.8	30.2 ± 7.8	30.5 ± 9.1	0.69
Smoker					0.19
Current	27 (56%)	16 (36%)	29 (40%)	72 (44%)	
Former	6 (12%)	5 (11%)	6 (8%)	17 (10%)	
Never	15 (31%)	23 (52%)	37 (51%)	75 (46%)	
ORIF					
Nail	24 (50%)	20 (45%)	34 (47%)	78 (48%)	0.91
Plate/screws	22 (46%)	19 (43%)	35 (49%)	76 (46%)	0.85
ESRD					
No	46 (96%)	44 (100%)	69 (96%)	159 (97%)	0.51
Yes	2 (4%)	0 (0%)	3 (4%)	5 (3%)	
Diabetes					
No	42 (88%)	37 (84%)	59 (82%)	138 (84%)	0.72
Yes	6 (12%)	7 (16%)	13 (18%)	26 (16%)	

**Table 2:** Fracture type by antibiotic group

Fracture	Cefazolin plus gentamicin plus tobramycin (N = 48)	Cefazolin plus gentamicin (N = 44)	Piperacillin-tazobactam (N = 72)	Total (N = 164)	P-value
Femoral shaft	4 (8%)	3 (7%)	4 (6%)	11 (7%)	0.91
Distal femur	10 (21%)	2 (5%)	14 (19%)	26 (16%)	0.056
Tibial plateau	2 (4%)	5 (11%)	4 (6%)	11 (7%)	0.38
Tibial shaft	22 (46%)	18 (41%)	28 (39%)	68 (41%)	0.75
Ankle/pilon	10 (21%)	16 (36%)	22 (31%)	48 (29%)	0.25

Most of the injuries were Gustilo-Anderson type IIIA (79%); 12% were type IIIB, 7% were unspecified type III, and only three patients (2%) had IIIC injuries, two of which were in the PT group. There was a statistically significant difference in the CG group with more type IIIB injuries (23%) and fewer type IIIA injuries (59%) (Table 3). 

**Table 3:** Gustilo-Anderson open fracture type by antibiotic group

Gustilo-Anderson type	Cefazolin plus gentamicin plus tobramycin (N = 48)	Cefazolin plus gentamicin (N = 44)	Piperacillin-tazobactam (N = 72)	Total (N = 164)	P-value
IIIA	44 (90%)	26 (59%)	61 (85%)	130 (79%)	0.009
IIIB	3 (6%)	10 (23%)	6 (8%)	19 (12%)	
IIIC	0 (0%)	1 (2%)	2 (3%)	3 (2%)	
Unspecified	2 (4%)	7 (16%)	3 (4%)	12 (7%)	

No statistically significant difference was identified for either deep or superficial SSIs among the three groups. There was also no difference in the rate of nonunion between groups (Table 4). Subanalysis of deep and superficial infections in DM and patients with ESRD showed no statistical difference in infection rates for these comorbidities; however, there was a trend towards more deep infections in diabetics (Table 5).

**Table 4:** Infection and nonunion rates by antibiotic group

	Cefazolin plus gentamicin plus tobramycin (N = 48)	Cefazolin plus gentamicin (N = 44)	Piperacillin-tazobactam (N = 72)	Total (N = 164)	P-value
Superficial infection	4 (8%)	5 (11%)	4 (6%)	13 (8%)	0.52
Deep infection	5 (10%)	9 (20%)	12 (17%)	26 (16%)	0.41
Nonunion	6 (13%)	9 (21%)	6 (9%)	21 (13%)	0.17

**Table 5:** Infection rates stratified by ESRD and DM by antibiotic group ESRD: end-stage renal disease, DM: diabetes mellitus

	All patients	Superficial infection	p-value	Deep infection	p-value
N	164	13		26	
ESRD			1.00		1.00
No	159 (97%)	13 (100%)		26 (100%)	
Yes	5 (3%)	0 (0%)		0 (0%)	
Diabetes			0.69		0.26
No	138 (84%)	12 (92%)		20 (77%)	
Yes	26 (16%)	1 (8%)		6 (23%)	

For Gustilo type IIIB injuries, neither time to definitive flap coverage nor flap type (free vs. local) was associated with a statistically significant difference in infection rates in our group. However, there was a trend towards deeper infections if definitive coverage was achieved >10 days after injury (Table 6).

**Table 6:** Infection rates by flap type and days until coverage for Gustilo-Anderson type IIIB injuries

	All IIIB patients	Superficial infection	p-value	Deep infection	p-value
N	19	3		6	
Days until coverage			1.00		0.32
0-10	13 (68%)	2 (67%)		3 (50%)	
>10	6 (32%)	1 (33%)		3 (50%)	
Flap type			1.00		1.00
Free flap	13 (68%)	2 (67%)		4 (67%)	
Local	6 (32%)	1 (33%)		2 (33%)	

## Discussion

Factors including soft tissue damage, wound contamination, vascular compromise, and fracture instability predispose type III open fractures to a multitude of complications, including infection, nonunion, and, in severe cases, amputation. Criticism has been voiced regarding the routine use of aminoglycoside antibiotics in type III open fractures, in part due to a lack of substantiated evidence outside of the original recommendations made by Gustilo and Patzakis based on culture isolates [1,2,5]. In conjunction with a well-documented risk of nephrotoxicity in this antibiotic class, especially in hemodynamically challenged trauma patients, the appropriateness of this prophylactic antibiotic regimen has been questioned [16-19]. Further problems with aminoglycosides include inappropriate or inaccurate dosing and a greater possibility of systemic complications when there is prolonged usage. Several groups have sought to find a safer and equally effective regimen. A previous study from our institution looking at the efficacy of injecting aqueous tobramycin into open fractures showed that the intervention group had a lower overall infection rate; however, these authors did not stratify infection by open fracture type and rather analyzed types I, II, and III open fractures together [20]. However, in a study of 85 patients, Shawar et al. have demonstrated that in type III open fractures, PT was superior to systemic tobramycin with cefazolin or clindamycin in preventing SSIs (4.3% vs. 27.5% at 30 days, p = 0.033) [22]. Similarly, in our study, there was no difference in PT prophylaxis compared to CG and CGT in preventing infections in type III open fractures of multiple bones. The authors included multiple fracture sites for two reasons. First, omitting some open fractures may have led to a bias, as perhaps the bones excluded had a higher infection rate. Second, this study, with some smaller numbers of fracture locations, shows efficacy in different locations.

A study in 2016 evaluated 126 type III open lower extremity fractures, 65 treated with a cephalosporin alone and 61 treated with a cephalosporin in conjunction with an aminoglycoside. They found no significant difference in the rate of SSI between groups (7% vs. 5%; p = 0.99) and a significantly higher incidence of AKI in the group treated with aminoglycosides (4% for the monotherapy group vs. 10% for the expanded gram-negative group; p = 0.05). This led them to conclude that prophylactic monotherapy with a cephalosporin may be sufficient and have a lower risk profile in the treatment of open grade III fractures of the lower extremity [19].

Another study evaluating 1,044 U.S. military personnel with open fractures sustained in Iraq and Afghanistan found no increased risk of osteomyelitis between those treated with narrow antibiotic prophylaxis (IV cefazolin, clindamycin, or amoxicillin-clavulanate) and those whose regimen included fluoroquinolones and/or aminoglycosides, although there was an increased risk of SSIs in the narrow-spectrum group. Interestingly, patients who received the extended-spectrum prophylaxis regimen subsequently had SSIs with organisms not susceptible to fluoroquinolones or aminoglycosides. These authors concluded that their results supported the current post-combat trauma antibiotic prophylaxis guidelines recommending the use of cefazolin monotherapy in all open extremity wounds, although Gustilo-Anderson type was not quantified [23].Dellinger and others have shown that short-duration prophylactic antibiotic administration is efficacious, and, in our series, we limited our use of PT to 24-72 hours after definitive fixation [24-26]. Our study emphasizes that there was no difference in PT, with a potentially lower adverse risk profile than aminoglycosides, and a short duration of 24 hours postoperatively, compared to the use of CG for longer durations.

Given the renewed discussion of appropriate antibiotic prophylaxis in type III open fractures and criticism regarding aminoglycoside use, there has been recent interest in alternative antibiotic therapies, namely, PT. While possessing a similar coverage spectrum to that of a cephalosporin and aminoglycoside, PT likely has a better safety profile than aminoglycosides, with the added benefit of being a monotherapy option. Furthermore, a study evaluating penetration characteristics of PT in cortical and cancellous tissues concluded that concentrations in bone tissues are sufficient to provide beta-lactamase activity, and that maintenance of concentration ratios in these tissues suggested efficacy in treating bone and joint infections by beta-lactamase-producing organisms [27].

Few studies directly assess the efficacy of PT in type III open fractures. Redfern et al. analyzed 72 patients with type III open fractures with the primary purpose of comparing rates of SSI at one year in those who were treated with PT versus CG. This study was performed at a level I trauma center, where it has been standard of care since 2009 to treat open type III fractures with PT monotherapy. Overall results demonstrated that both prophylaxis regimens demonstrated comparable results with respect to primary and secondary outcomes. The one-year SSI rate was 32.4% (12/37) for the CG group and 31.4% (11/35) for the PT group (P =1.000). There were no significant differences between the groups in rates of 30-day SSI or nonunion at one year. The major criticism of this study was its small sample size and that it was underpowered to detect statistically significant differences between antibiotic regimen outcomes. Nevertheless, the results did suggest PT may be an efficacious alternative to the use of CG for type III open fracture prophylaxis [28]. In addition, O’Connell et al. evaluated 120 type III open fractures, 97 treated with PT, and 23 treated with guideline-concordant therapy defined as first-generation cephalosporin with or without an aminoglycoside. Their multivariable analysis demonstrated no difference in infections (p = 0.096) and no differences in adverse events, including delayed union, nonunion, and development of *Clostridioides difficile* [29].Our study examined 164 type III open fractures (48 (29%) in the CGT group, 45 (27%) in the CG group, and 72 (44%) in the PT group), and although these numbers may not be sufficient to prove clinical significance, they are indeed consistent with other published results and may be the largest comparative series in the literature. 

There are several limitations to our study. The necessity to use CPT codes and to manually filter for type III injuries, combined with some inherent subjectivity of the Gustilo-Anderson classification system, as has been previously demonstrated, introduces the possibility for type III injuries to be unintentionally excluded from the study [30]. Brumback showed that the average agreement using the Gustilo-Anderson classification was only 60%; however, this was used by the treating surgeons and is the most widely used and understood classification, so this was employed in our study [30]. The authors carefully read through all operative notes but felt that the treating surgeon at the initial operation had the best opportunity to classify the open nature of these injuries. Furthermore, only certain common fractures were included, and our search was not inclusive of all possible open fractures. Another limitation was the non-standardized, surgeon-discretion use of injected tobramycin, as per the preference of several surgeons. To account for this, those receiving injected tobramycin were placed into their own treatment group, although this did reduce potential treatment group sizes. We did not include a variable to adjust for time; PT was used primarily in 2016 and later, and we did not have any other practice changes at our institution that would have confounded these results. Although we did not directly compare adverse events between the groups in our study, based on the literature, we would expect less nephrotoxicity in the PT group. Lastly, the rarity of the injury of interest makes collecting numbers high enough to draw statistically significant conclusions difficult. As it is a rare event, this study provides the largest sample in the literature. The statistical trend is felt to draw valuable clinical significance. 

## Conclusions

Our data support the use of PT as a short-term monotherapy antibiotic prophylaxis for type III open fractures. The trend in our data suggests no difference in SSIs or nonunion when compared to traditional regimens, with the benefit of a lower toxicity profile. We feel that the use of PT monotherapy is a reasonable alternative to a first-generation cephalosporin plus an aminoglycoside in type III open fractures. There is insufficient evidence to suggest that PT is favorable over other prophylactic regimens, and it also cannot be concluded that there are lower nephrotoxic effects with PT based on these data. The authors do feel that PT is a safe option for antibiotic prophylaxis and recommend coordinating with both the infectious disease and pharmacy teams at a given institution to develop appropriate prophylactic antibiotic protocols at a given institution. 
